# Supplementary material for: Comparative genomics provides new insights into the diversity, physiology, and sexuality of the only industrially exploited tremellomycete: Phaffia rhodozyma
Source: BMC Genomics. 2016 Nov 9;17:901. doi: 10.1186/s12864-016-3244-7 (PMC5103461; doi:10.1186/s12864-016-3244-7)
Supplement: Additional file 6: — List of orphan genes with links to PFAM (related to Additional file 1: Table S1). (ZIP 1428 kb) [file 12864_2016_3244_MOESM6_ESM.zip › PFAM_UNIQUE/G00561_P.html]

#                                                                            --- full sequence --- -------------- this domain -------------   hmm coord   ali coord   env coord
  
# target name        accession   tlen query name           accession   qlen   E-value  score  bias   #  of  c-Evalue  i-Evalue  score  bias  from    to  from    to  from    to  acc description of target
  
#------------------- ---------- ----- -------------------- ---------- ----- --------- ------ ----- --- --- --------- --------- ------ ----- ----- ----- ----- ----- ----- ----- ---- ---------------------
  
G00561\_P             -            352 DUF1777              PF08648.7    180       1.9    6.9   6.9   1   2       2.8   8.4e+02   -1.8   0.0   122   139    75    93    66    97 0.79 - Protein of unknown function (DUF1777)  
G00561\_P             -            352 DUF1777              PF08648.7    180       1.9    6.9   6.9   2   2     0.052        16    3.9   8.3    42    96   159   215   136   233 0.47 - Protein of unknown function (DUF1777)  
G00561\_P             -            352 DUF605               PF04652.11   380      0.13   10.2   2.4   1   1    0.0031      0.19    9.7   2.4   142   269   126   260   121   277 0.56 - Vta1 like
